# Evaluation of *FLT3-ITD* Mutations and *MDR1* Gene Expression in AML Patients 

**DOI:** 10.30699/IJP.2022.543485.2776

**Published:** 2022-09-20

**Authors:** Ehsan Yazdandoust, Mohammad Hadi Sadeghian, Seyyede Fatemeh Shams, Hossein Ayatollahi, Yasaman Saadatpour, Payam Siyadat, Maryam Sheikhi, Monavvar Afzalaghaee

**Affiliations:** 1 *Blood Transfusion Research Center, High Institute for Research and Education in Transfusion Medicine, Tehran, Iran*; 2 *Cancer Molecular Pathology Research Center, Mashhad University of Medical Sciences, Mashhad, Iran*; 3 *Mashhad University of Medical Sciences, Mashhad, Iran*; 4 *Department of Hematology, School of Allied Medical Sciences, Iran University of Medical Sciences, Tehran, Iran*; 5 *Department of Community Medicine, Mashhad University of Medical Sciences, Mashhad, Iran*

**Keywords:** Acute myeloid leukemia (AML), Cuplike morphology, FLT3-ITD, Gene expression, Real-Time PCR (RT-PCR)

## Abstract

**Background & Objective:**

Acute myeloid leukemia (AML) is a hematopoietic malignancy caused by genetic abnormalities. Currently, molecular and genetic factors are routinely used as diagnostic and prognostic markers. FLT-3 is one of the most known diagnostic factors in AML. *MDR1* gene belongs to the ATP binding cassette family; it is known as one of the chemotherapy-resistant causes of AML. We aimed to study *FLT-3ITD* mutations and their association with *MDR1 *gene expression in AML individuals.

**Methods:**

For investigation, 80 AML individuals and 20 healthy controls were selected. This study was done in the Cancer molecular Pathology Research Center of Mashhad University of Medical Sciences (MUMS), Iran during 2017-2019. *FLT3-ITD* mutation was assessed by polymerase chain reaction (PCR); Real-time quantitative PCR was performed to measure the amount of *MDR1* gene expression. Bone marrow and blood smears of patients were evaluated in terms of morphology. SPSS 16.0 was used for data analysis.

**Results:**

*FLT3-ITD* mutation and *MDR1* overexpression were found in 18.8% and 23.8% of AML patients, respectively. Statistical analysis did not show any relationship or association between these two markers. Cuplike morphology was observed in blast cells in 21.25% of AML cases, which was associated with the presence of *FLT3-ITD* mutation.

**Conclusion:**

*FLT-3* and *MDR1* function independently. Survival studies to determine the exact role of *MDR1 *overexpression in drug resistance issues would be suggested.

## Introduction

Acute myeloid leukemia (AML) is an aggressive and proliferative disorder involving hematopoietic cells (1, 2). It comprises 75-80% of acute leukemia cases in adults and 15% in children (3). Leukemia is the result of genetic abnormality in the hematopoietic precursors. The genetic instability causing the disease is very applicable in the diagnosis, prognosis determination, and even the treatment protocol selection. 

WHO has classified AML based on the molecular and cytogenetic findings of malignancy. *FLT3* mutations, including ITD (internal tandem duplications) and TKD subtypes, are among the most effective and prevalent genetic alterations. ITD type is more prevalent, and TKD is rare. *FLT3-ITD* mutations are detectable in 33% of AMLs, and mutation of the tyrosine kinase domain (TKD) occurs in ~ 10% of AML patients; *FLT3* mutations are associated with decreased overall survival and higher rates of relapse and are more common in normal karyotype ones (4, 5). Its prognosis is affected by mutant allele level and gene expression and the presence of co-existing mutations (4, 6).

Gene expression evaluation is another effective molecular factor that affects AML prognosis. The results of this assay will characterize the disease's clinical outcomes. The human multidrug resistance gene (MDR1) abnormality is reported in many cancers (7). It belongs to the ATP-binding cassette superfamily (8), responsible for toxins removal from cells. As the gene name suggests, its overexpression is associated with poor prognosis and chemotherapy resistance outcomes (8, 9). The gene product affects cells by changing drug pharmacokinetics; this function is very important in fighting against cancerous cells (9). Evaluation of *MDR1 *expression and *FLT3-ITD* mutations in AML patients is important, and it has been shown that patients with both *FLT3-ITD* mutations and MDR1 overexpression have poorer overall survival (6).

We searched many studies in the molecular field of AML, but limited papers have evaluated the relationships between the amount of *MDR1* gene expression and the presence of *FLT3* mutation; the present study was designed due to a lack of sufficient information in the cited area. 

## Material and Methods

**Patients**

This case-control study was performed in the Cancer Molecular Pathology Research Center and hematology lab of Ghaem Hospital (Mashhad University of medical sciences) from October 2017 to March 2019. In this survey, 80 AML patients, including 44 men and 36 women, from different subtypes (M1, M2, M3, and…) were examined; twenty healthy individuals were considered as a control group. Cases with secondary myelodysplastic syndrome (MDS), secondary AML, and Down syndrome were excluded from the patient group. AML was diagnosed according to the French-American-British (FAB) classification, Immuno-phenotypic characteristics, and laboratory findings; the AML subtype was also classified according to FAB classification.

Bone marrow and peripheral blood specimens were collected in EDTA container tubes. Patients' peripheral blood and bone marrow smears were carefully evaluated for cell morphology. Blast and differential counts were performed, and the blasts' morphology and other cells were considered.

**RNA Extraction**

Total RNA was extracted from mononuclear cells of the samples using the TriPure Isolation Reagent Kit (Roche Diagnostic, Germany); nanodrop was used for evaluating the extracted RNA. Patient RNA samples were loaded in the agarose gel with loading dye; approximately the 28S rRNA band should be twice as intense as the 18S rRNA. Extracted RNA was stored at -80 °C until the test time.

**c-DNA Synthesis**

Complementary DNA (cDNA) synthesis was done by Revert Aid TM cDNA Synthesis Kit (Fermentas, Germany); cDNA was used as a template for PCR amplification for FLT3/ITD mutation identification and MDR1 gene expression. 

**Analysis of the Flt3/ITD**

Exons 11 and 12 of the FLT3 were ampliﬁed by polymerase chain reaction (PCR) using the following primers: 5'TGGTGTTTGTCTCCTCTTCATTGT-3` as the forward and 5`-GTTGCGTTCATCTTTCAAA-3` as the reverse one. The PCR was done in 10x PCR buffer II, MgCl_2_, dNTPs, Taq DNA polymerase (CinnaGen, Iran), cDNA (1:50). 

**Fig. 1:**
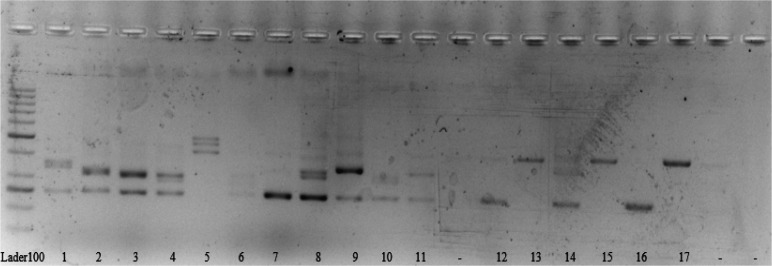
PCR for the *FLT3/ITD* samples from AML patients on agarose gel 4%. Two Band samples and a heavier band than 200 base per were positive for *FLT3-ITD* mutation. Single-band samples, such as Nos. 7 and 16 were negative for the mutation; they form bonds of about 200 base pairs. Mutant specimens formed a heavier band than 245 base pairs. Also, the same result was seen in 8% polyacrylamide gel

Reaction conditions, including denaturing, annealing, and extension steps, were performed at 94°C for 30 seconds, 61°C for 30 seconds, and 72°C for 30 seconds for 35 cycles in the Thermal Cycler (Applied Biosystems). Also, the initial denaturation step was 5-minute at 95°C, and the ﬁnal extension was 72°C for 5 minutes. Products were electrophoresed in 8% polyacrylamide gel and 4% agarose gel. The emergence of a single band in the 245 bp size indicated no mutation (wild type); a double band indicated a mutation in the FLT3 gene of the ITD type (Figure1).

**Real-time Polymerase Chain Reaction (RQ-PCR)**

The real-time qPCR technique with the SYBR Green method was performed to evaluate the MDR1 mRNA expression in the AML and control samples. cDNA was used for this procedure. Glyceraldehyde 3-phosphate dehydrogenase (GAPDH) gene was considered the reference one; it was applied for data normalization to correct RNA quality and quantity variations. This procedure was performed by SYBR Premix Ex Taq TM II kit (Takara, Japan) on an ABI thermos cycler (One-Step, USA). Also, a 5-fold serial dilution of GAPDH c-DNA and MDR1 c-DNA was performed to determine the efficiency before the procedure. 

All samples were performed in Triplicate. The reaction mixture contains 4 μL of cDNA template, 0.4 μL of each primer in Table 1; they were employed according to the kit's protocol (Takara, Japan). The amplification process was as follows: denaturation at 95°C for 2 minutes followed by 40 cycles at 95°C for 20 seconds, 59°C for 1 minute, and final elongation 72°C for 1 minute. The 2^-ΔΔCT^ method was performed for relative gene expression calculation. The specificity of products was confirmed by the melt curve.

The mean expression of the *MDR1* gene in the control group was considered a cut-off value (1.5). Samples with the *MDR1* expression higher than 1.5 were categorized as overexpressed cases (MDR1 positive), and *MDR1* expressions less than 1.5 were known as negative.

**Statistical Analysis**

Statistical analysis was conducted using the statistical program SPSS software, version 16 (SPSS Inc., Chicago, Ill., USA). The association between the levels of MDR1 m-RNA expression with FLT3-ITD mutations was determined by Fisher's exact test. The association was considered statistically significant when P-value ≤ 0.05 was achieved.

**Table 1:** The primers used for RQ-PCR

**5`-AGGCCGCTGTTCGTTTCCTTTAGGTC-3`**	**MDR1-Forward**
**5`-AGATCCATTCCGACCTCGCGCTCCT-3`**	MDR1-Reverse
**5`-GCCCCAGCAAGAGCACAAGAGGAAGA-3`**	GAPDH-Forward
**5`-CATGGCAACTGTGAGGAGGGGAGATT-3`**	GAPDH-Reverse

**Table 2:** Patients' molecular data based on FAB classification and recurrent genetic mutation

FAB subtype	Frequency	Mutation	Frequency
M0	%7.5 (6)	t (8;21)	8.8% (7)
M1	%11.3 (9)	t (15;17)	27.5% (22)
M2	%26.3 (21)	Inv16	2.5% (2)
M3	%22.5 (18)	**Nonrecurrent abnormalities**	61.3% (49)
M3v	%7.5 (6)	FLT3-ITD^+^	18.8% (15)
M4	%10 (8)	MDR1^+^	23.8% (19)
M4eo	%1.3 (1)	**Total**	**80**
M5	%10 (8)		
M6	%1.3 (1)		
M7	%1.3 (1)		
Unknown	%1.3 (1)		
Total	**80**		

## Results

**Patients Information**

**Fig. 2:**
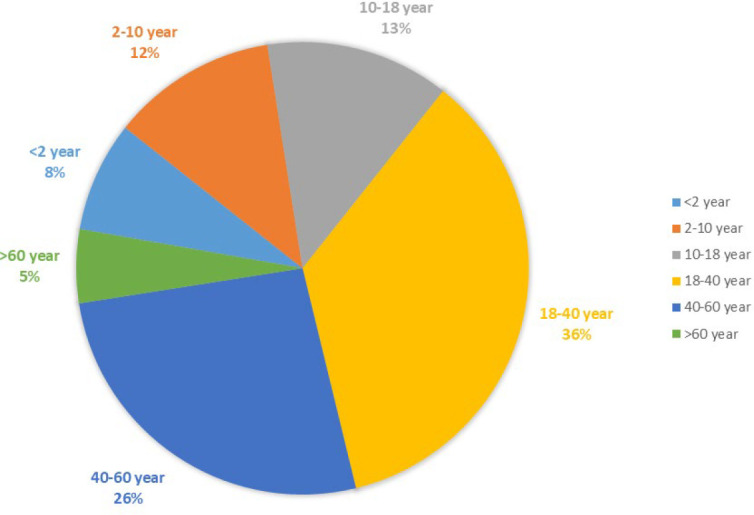
Distribution of age group in the studied AML patients

The samples belonged to the archive of the Cancer Molecular Pathology Research Center of Mashhad University of Medical Sciences, Iran. Of the studied individuals, 55% (44) were male, and 45% (36) were female. The age range was 2-60 years; the mean age of studied patients was 38 years (Figure 2). *FLT3-ITD* was detected in 15 out of 80 (18.8%); *FLT3-ITD* frequency was equal in both groups of patients, including recurrent mutation and non-recurrent ones. *FLT3 ITD* mutation was more frequent in M3, M4, and M5 subtypes of AML. Molecular data of patients have been summarized in Table 2. Mean hematologic indices of studied patients were as follows, hemoglobin 8.22±2.3 gr/dl, white blood cell count: 34.86±35×10^3^/μL, platelet count: 58.39/dl±47.9 ×10^3^/μL, and blast: 63.28±23%. The blast cell count in our patients with *FLT3-ITD* gene mutation was higher (74.5% vs. 50.1%, *P*=0.003).

The mean *MDR1* gene expression level in the control group was 1.5±0.56 and in AML patients was 4.64±14.41, ranging between 0.003 and 61.141. The average *MDR1* gene expression level was not statistically significant between *FLT3-ITD* positive patients and group of patients without *FLT3-ITD* mutation (5.2±12.63 vs. 3.6±16, respectively; *P*=0.218).

*MDR1* overexpression was found in 23.8% (19 individuals) of AML cases; overexpression was observed in 33% of M0, 33% of M1, and 25% of M4/5 subtypes. Besides, *MDR1 *gene overexpression was found in 26.5% of AML individuals without recurrent mutations and 19.3% of cases with recurrent mutations. This finding was not statistically significant (*P*=0.5). Evaluation of AML patients with recurrent mutations showed that the number of expressed gene was lower in cases with good prognosis mutations such as t (8;21), t (15;17), inv (16) in comparison to the patients, who did not have any good prognostic markers, but This finding was not statistically significant (*P*=0.339). 

No direct or reverse correlation was found between *FLT3-ITD* mutation and *MDR1* gene expression (*P*=0.499) by the exact fisher's exact test; in fact, they are not involved in their pathogenesis.

**Fig. 3:**
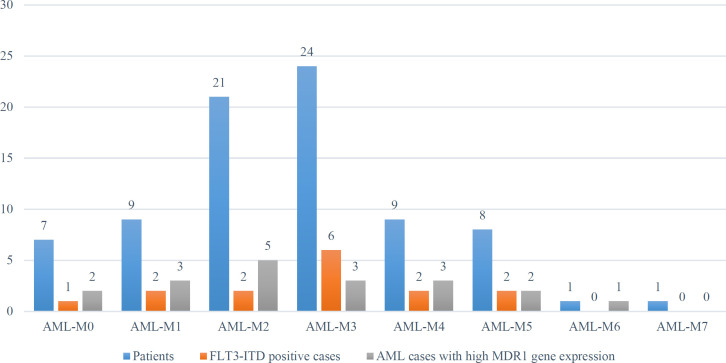
Frequency distribution of AML subtypes and *FLT3-ITD* positive cases and cases with high *MDR1* gene expression in studied AML patients

**Fig. 4:**
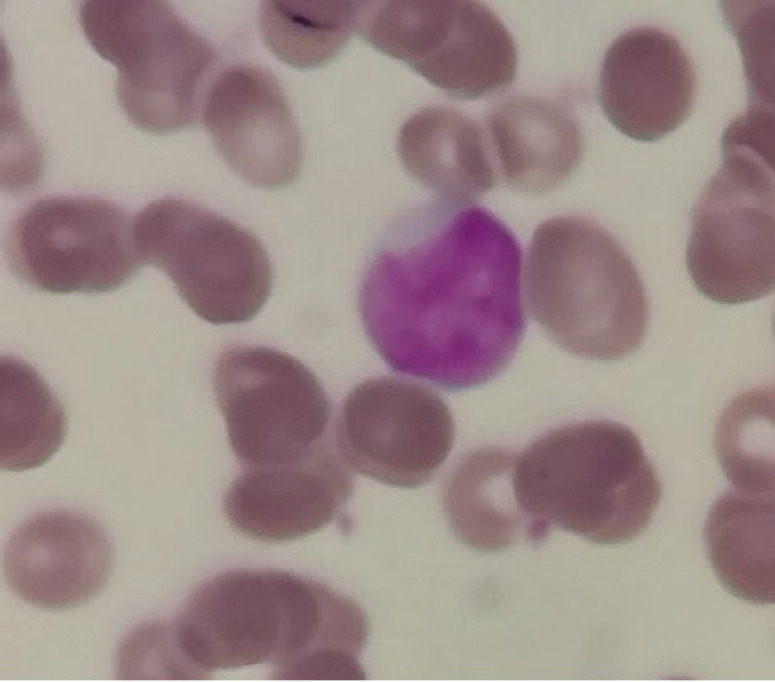
Cuplike morphology in patients in the present study, which is more common in the cases with *FLT3-ITD* mutations

Bone marrow and peripheral blood smear examination showed cuplike or fish mouth blasts in 17 out of 80 cases (21.25%). *FLT3-ITD* mutation was observed in 11 patients with cuplike morphology (11 of 17 patients (64.7%)). A strong correlation was found between *FLT3-ITD* mutation and cuplike morphology. This morphology was also detected in rare negative cases of *FLT3* mutation (1-5%). Cuplike morphology is more possibly seen in peripheral blood blasts. Among 17 cases with this morphology, cuplike blasts were detected in the peripheral blood of 9 cases and were not observed in the bone marrow; so, to diagnose this morphology, a peripheral blood smear is preferable to bone marrow aspiration (Figure 3 and Figure 4).

## Discussion

In this study, we evaluated two prognostic factors including *FLT3-ITD* mutation and *MDR1* gene expression in AML cases. Previous studies about the relationship between these two factors are limited. The results of the present study showed no statistical relationship between these two prognostic markers; actually, they function independently (*P*=0.499).

*FLT3* mutations are prevalent among AML patients; approximately, they are found in 25 -35% of AML cases. *FLT3-ITD* is the most common type among *FLT3* mutations family, which is detectable in 20-27% of adults and 10-12% of children (10-14). ITD type frequency has been reported by Yuslina Mat Yusoff in Malaysia (16.1% among 199 normal karyotype AML individuals) (15) and Bhattacharyya study (14.54% of the Indian patients) (16); their reported rate is similar to the finding of the present study (18.8% in both groups of normal karyotype and recurrent mutations). This similarity seems important according to various ethnicity and geographical area; it indicates the importance of *FLT3* in the diagnosis procedure regardless of differences. The cited mutation was detected in all AML subgroups of our patients; it was mostly associated with M3, M4, and M5 leukemia. This finding was consistent with other studies (12, 14, 17-19). Regarding the effect of sex on FLT3-ITD mutation, the prevalence was almost equal in both genders, although there was a comparative advantage with males, which is supported by other studies (20, 21). 

As it was mentioned in the previous paragraphs, statistical analysis demonstrated that *FLT3-ITD* and *MDR1* genes are two independent factors, and none of them affect the others. Nasilowska *et al.* studied the *FLT3-ITD* influence on MDR1, MRP1 and BCRP mRNA expression, which are related to AML progression. They reported higher expression of MDR-1 in patients who were negative for *FLT3-ITD*; they believed that more investigations are required to find any correlations (22). 

Galimberti *et al.* published a paper in 2003 which claimed a lack of relation between FLT3-ITD and MDR-1(23); Xu also reported the same results in terms of FLT3 relation to *MDR1* (24). Despite the whole of the above studies, Kassem and coworkers discovered a significant occurrence of FLT3 mutation in overexpressed MDR-1 AML individuals (2). It seems that the results of heterogeneity are due to the *MDR1* gene role in the chemotherapy response; all researchers selected AML cases randomly, but in the following *MDR1* overexpressed group was resistant to chemotherapy. Different categorizations may cause different results. 

 Comparing the amount of *MDR1* mRNA expression between the two groups under study demonstrated significantly higher expression in AML patients. Another paper by Kassem *et al.,* which studied 100 AML individuals, also reported having higher expression of *MDR1* mRNA in the AML group compared to healthy controls (2). This finding has been approved by an Egyptian cohort (25), and Xu *et al.* study, too (24).

Many studies evaluated the correlation between the *MDR1* gene and other AML prognostic markers, such as NPM-1, WT-1, etc., but the number of performed studies on *MDR1* is limited. According to the *MDR1* gene product function, most of them analyzed the impression of *MDR1* on the patients' survival (survival study).

Also, in this study, patients' peripheral blood and bone marrow smears were evaluated using Giemsa and Wright–Giemsa dye. We found out that *FLT3-ITD* mutation and cuplike morphology (fish mouth blast) are associated with each other; this finding has been reported by other studies, too (26, 27). Our study showed a greater relationship between these two factors. In our study, we found a significant association between *FLT3-ITD* mutations and bone marrow blast count, consistent with other studies (28-30).

Also, some studies have indicated that *npm1* and *FLT3-ITD* mutations are related to cuplike morphology and may predict a bad prognosis (31-33), but Kroschinsky *et al.* believed this morphology could not affect the response to treatment or survival (32). The limitation of the present study was the small number of the participants, which affected the generalizability of the results. To obtain more reliable results, a larger statistical community is recommended.

## Conclusion

Finally, no relationship was found between FLT3-ITD mutation and *MDR1* overexpression. Also, we found a strong relationship between *FLT3-ITD* mutations and cuplike morphology, which can guide us to find and detect these mutations in AML patients. No relationship was also found between cited morphology and *MDR1* gene expression.

Researchers have announced that *MDR1* can predispose a patient's response to disease. It is important to mention that *mrd1* polymorphisms have different clinical outcomes. As it was earlier mentioned, it is worth to design a survival study to determine the effect of *MDR1* overexpression on clinical response and overall survival rate of the patients. 

##  Ethics Approval

The present study was approved by the ethics committee of Mashhad University of Medical Sciences (ethic code: IRMUMS.fm.REC.1394.536).

## Authors' Contributions

Contributor 1, 2 designed the research. Contributor 1, 2, 4, 5, 7 carried out the experiments. Contributor 3, 6 wrote the manuscript with support from Contributor 1, 2. Contributor 3, 8 performed the statistical analysis of the date. Contributor 1, 4 worked out almost all the technical details and performed the calculations for the suggested experiments. All authors participated in discussion of the results and made comments and suggestion on the manuscript and contributed to the research. 

## Conflict of Interest

The authors declared no conflict of interest.

## Funding

This study was fully sponsored by Mashhad University of Medical Sciences, Mashhad, Iran.
